# What Matters Most: The Top 10 Child and Adolescent Cancer Research Priorities in Australia

**DOI:** 10.1111/hex.70689

**Published:** 2026-05-16

**Authors:** Gayani De Silva, Eden G. Robertson, Alison Bowers, Clarissa Schilstra, Sheila K. Patel, Maria C. McCarthy, Jordana McLoone, Jason D. Pole, Ursula M. Sansom‐Daly, Sabina Oppelaar, Joanne Cummings, Natalie Bradford

**Affiliations:** ^1^ Cancer & Palliative Care Outcomes Centre, Centre for Healthcare Transformation, Faculty of Health Queensland University of Technology Brisbane Queensland Australia; ^2^ Behavioural Sciences Unit, School of Clinical Medicine, UNSW Medicine & Health, Discipline of Paediatrics and Child Health, Randwick Clinical Campus, UNSW Sydney Randwick New South Wales Australia; ^3^ Kids Cancer Centre, Sydney Children's Hospital Randwick New South Wales Australia; ^4^ Representation With Childhood Cancer Experience on the Child and Adolescent Cancer Priority Setting Partnership Sydney New South Wales Australia; ^5^ Parent Representative on the Child and Adolescent Cancer Priority Setting Partnership Melbourne Victoria Australia; ^6^ Department of Medicine University of Melbourne Melbourne Victoria Australia; ^7^ Children's Cancer Centre, Royal Children's Hospital Parkville Victoria Australia; ^8^ Murdoch Children's Research Institute Parkville Victoria Australia; ^9^ Department of Paediatrics University of Melbourne Melbourne Victoria Australia; ^10^ Queensland Digital Health Centre, Centre for Health Services Research, Faculty of Health, Medicine and Behavioural Sciences University of Queensland Brisbane Queensland Australia; ^11^ Sydney Youth Cancer Service, Prince of Wales Hospital Randwick New South Wales Australia; ^12^ School of Nursing, Faculty of Health Queensland University of Technology Brisbane Queensland Australia; ^13^ Anglicare Sydney New South Wales Australia; ^14^ Viertel Cancer Research Centre, Cancer Council Queensland Brisbane Queensland Australia

**Keywords:** adolescent, cancer, child, priority setting partnerships, research priorities

## Abstract

**Background:**

Children and adolescents diagnosed with cancer and their families have unique medical and psychosocial needs. Addressing these requires research centred on issues most relevant to them. Currently, Australia lacks a research agenda grounded in lived experience and clinical expertise to guide inquiry into child and adolescent cancer.

**Objective:**

To identify the top 10 research priorities for child and adolescent cancer in Australia.

**Methods:**

We conducted a James Lind Alliance Priority Setting Partnership involving two national online surveys and an online workshop. Individuals diagnosed with cancer before age 19, caregivers, and health professionals providing care were involved in the process.

**Results:**

In Survey 1, 229 respondents (41 patients/survivors, 118 caregivers, 70 professionals) submitted 602 in‐scope research questions. These were grouped and refined into 49 summary questions and verified as unanswered through literature reviews. In Survey 2, 474 respondents (32 patients/survivors, 289 caregivers, 139 professionals) selected and ranked the questions most important to them, narrowing the list. The top‐ranked 19 questions were then discussed in a workshop with 27 participants (7 survivors, 9 caregivers, 11 professionals) to reach consensus on the final top 10. Priorities span treatment, survivorship, psychosocial support, and service delivery.

**Conclusions:**

These priorities provide the first step toward establishing a child and adolescent cancer research agenda in Australia that reflects both lived experience and clinical expertise. They call for future research into safe and personalised care across the trajectory, delivered with equality using culturally safe approaches. Collaboratively advancing these priorities will accelerate translation of evidence into meaningful outcomes.

## Patient or Public Contribution

Patient and public involvement is at the core of our chosen methodology and central to our Priority Setting Partnership from inception. We included childhood cancer survivors, caregivers, health professionals who provide care for them, and representatives from community support organisations as Steering Group members and partners. Together, the Steering Group co‐developed and refined the project scope to ensure relevance, designed and reviewed survey questions and recruitment materials, and supported survey participant recruitment through their networks. They played a key role in interpreting survey results and workshop data and advised on the framing and language used to disseminate findings, thus ensuring the outputs were accessible, meaningful, and resonant with affected communities. They also participated as the long‐term advisory panel overseeing the project, monitoring the fairness and transparency in decision‐making. Additionally, two individuals with lived experience (from the Steering Group) contributed to the writing and editing of this manuscript. In accordance with these contributions, they are included as co‐authors.

**Abbreviations**
ANZCHOG: Australian and New Zealand Children's Haematology/Oncology Group
JLA: James Lind Alliance
PSP: Priority Setting Partnership
SG: Steering Group

## 1 Introduction

Child and adolescent cancer remains one of the most serious health challenges facing young Australians. In 2024, nearly 1,280 children and adolescents (aged 0 ‐ 19 years) were diagnosed with cancer in Australia alone, underscoring the ongoing demand for specialised age‐appropriate oncology services [1]. Survival outcomes have steadily improved in Australia, with 5‑year disease‑free survival rates now reaching up to 87% [2]. However, the burden extends far beyond survival, with approximately 95% of child and adolescent cancer survivors experiencing long‑term adverse effects stemming from both the disease and its treatments [3], with consequences that extend across families, health systems, and the broader economy. To optimise outcomes and minimise these lifelong impacts, children and adolescents must receive timely, age‑appropriate, evidence‑based care. This care should address the full spectrum of medical, developmental, and psychosocial needs throughout the cancer journey. Conducting relevant research is essential to address these needs, enhance patient experiences and outcomes, and inform the development of evidence‐based clinical practice guidelines and health policies.

In both Australia and globally, health research agendas are most often shaped by researchers and funding organisations [4, 5, 6]. While these groups play a critical role in advancing science, their perspectives alone can result in a disconnect between the research undertaken and the needs of those most affected, including patients, caregivers, and the health professionals providing care [7, 8]. Such misalignment can reduce the relevance and impact of research, hinder clinical uptake and knowledge translation, and lead to inefficient use of time and resources, ultimately missing opportunities to improve care [9, 10]. A targeted research agenda that addresses evidence gaps and is grounded in the lived experiences of patients, families, and health professionals can promote efficient resource allocation, drive meaningful outcomes, and support economic sustainability [11, 12, 13]. To address this need, priority setting partnerships provide a structured approach that brings together diverse stakeholders to identify and prioritise key research questions grounded in the lived experiences of those most impacted by the disease [14].

The global childhood cancer research landscape is marked by substantial inequities in investment, access to clinical trials, and the strength of research ecosystems [15]. Despite major advances, these disparities are evident not only in low‐ and middle‐income countries, but also within high‐income settings, where unequal access to high‐quality, research‐driven care persists [16]. This evidence highlights the need for innovative and coordinated research strategies to accelerate progress in paediatric oncology. In response, the United Kingdom and Canada have undertaken structured national priority‐setting processes to identify key research priorities in childhood and adolescent cancer, shaped by patients, families, clinicians, and researchers, to shape context‐specific childhood cancer research agendas [17, 18, 19, 20, 21]. In contrast, the Australian context remains underexplored, with no equivalent national prioritisation efforts to guide future childhood cancer research.

Australia has a unique geography, a diverse population, and notable differences in health care access and availability, which shape families’ experiences of child and adolescent cancer. These factors influence treatment choices, communication styles and needs with patients and families, and engagement with support systems, which may create distinct contextual differences in research needs and priorities across the country. Several research priority initiatives related to cancer have been conducted in Australia. These focus on adult cancer survivorship and general practice, adolescent and young adult cancer research, and a review of research related to Aboriginal and Torres Strait Islander children with cancer [22, 23, 24, 25, 26, 27]. These efforts are restricted to specific subfields and have not considered the full scope of child and adolescent cancer across all age groups, cancer types, stages of care, and the health system. The absence of a targeted research agenda for child and adolescent cancer with a holistic approach highlights a critical gap in the current research landscape. To address this, we engaged individuals with lived experience of child and adolescent cancer and health professionals serving this community to collaboratively identify and prioritise unanswered research questions for child and adolescent cancer.

## 2 Methods

**Figure 1:**
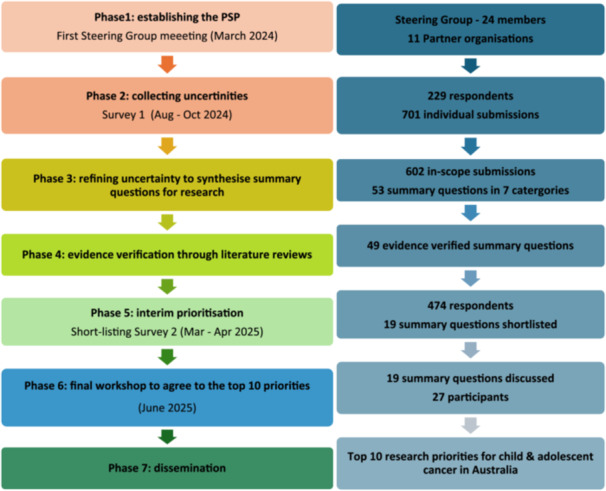
A schematic overview of the Priority Setting Partnership process.

We undertook a James Lind Alliance (JLA) Priority Setting Partnership (PSP), following the well‐established and standard methodology used across all JLA PSPs [28]. This involved the following phases 1); establishing the PSP and steering committee to guide the PSP process 2); collecting unanswered questions framed as researchable issues (uncertainties) (Survey 1) 3); refining uncertainties to synthesised summary questions for research 4); reviewing literature to verify uncertainties as unanswered 5); shortlisting uncertainties through an interim prioritisation survey (Survey 2) 6); workshop to reach consensus on the final top 10 priorities, and 7); dissemination of priorities. A schematic overview is provided in Figure 1. The PSP protocol is available online [29]. We report our PSP according to the reporting guidelines for priority setting of health research guidelines [30].

### 2.1 Phase 1: Establishing the Priority Setting Partnership

A team of researchers partnered with JLA to establish the Australian Child and Adolescent Cancer PSP in March 2024. This phase involved identifying the national Steering Group (SG) and partner organisations (see acknowledgements) and defining the scope of the PSP. The SG collaborated to set the scope, promote the project, guide the data collection, and provide expert input in analysis, while overseeing the project throughout the study period. The SG included 24 members representing the following stakeholder categories: adult survivors of child and adolescent cancer (*n* = 2), parents of a child diagnosed with cancer (*n* = 7); a range of professionals reflecting the multidisciplinary nature of the care of children with cancer including: paediatric oncologists (*n* = 2), paediatric psychologists (*n* = 2), nurses (*n* = 4), genetic counsellors (*n* = 2) representatives from charities/funding organisations (*n* = 3) and researchers (*n* = 2). The JLA Facilitator (SK), an independent representative from the UK, chaired the meetings. As someone who is not a health professional, researcher, or person with lived experience and from Australia, SK was well‐positioned to guide the process impartially and ensure fair, transparent discussion across all participants. We collaborated with partners, primarily child and adolescent cancer charities and professional networks. Overall, we had 11 partner organisations supporting recruitment and dissemination.

### 2.2 Scope of the PSP

The scope was intentionally defined as broad to include questions for research across the continuum of cancer care: prevention, diagnosis, treatment, survivorship, and beyond. We considered all types of child and adolescent cancer and brain tumour diagnosed between 0 and 19 years to reflect the spectrum of conditions and ages treated in Australian tertiary children's cancer centres.

The following individuals were eligible to participate in the PSP:

i. Individuals (including children and adolescents) who were diagnosed with any type of cancer or brain tumour as a child or adolescent before their 19th birthday
ii. Caregivers (parents/relatives/kinship) of a child or adolescent who was diagnosed with cancer before their 19th birthday
iii. Health or social care professionals who provide cancer care to children or adolescents diagnosed with cancer.

To ensure an Australian focus, respondents were required to reside in Australia and have received or provided cancer treatment in Australia.

### 2.3 Phase 2: Collecting Uncertainties

We collected uncertainties using an online national survey (Survey 1) developed and delivered using Qualtrics, a secure online survey platform. Survey 1 was developed by the lead author (GD) and refined by the SG. The survey allowed respondents to submit up to five questions on child and adolescent cancer that they considered important and unanswered, and included a separate section to collect demographic data to ensure stakeholder representation. The SG recruited pilot participants through their peer and professional networks. We piloted the wording and design of Survey 1 with 12 survivors of child and adolescent cancer (including three children under 18 years), 13 parents, and 17 health professionals outside the SG, with representation from Queensland, Victoria, New South Wales, and South Australia. We made minor refinements to the survey following participant feedback from the pilot and then Survey 1 was kept live for 3 months. Survey promotion occurred through the PSP website [31], social media, and professional, peer, and partner networks. Throughout recruitment, the SG monitored respondent representativeness and targeted efforts to engage underrepresented groups, such as young patients and those from the Northern Territory.

### 2.4 Collecting Questions From Children and Adolescents

The survey was specifically designed to facilitate meaningful participation from children and adolescents affected by cancer, having a separate child‐friendly subsection to be completed with their caregivers’ help and consent. Parents were contacted through partner organisations and personal networks to support participation, and child‐friendly promotional videos with animations were used to clearly explain the study. To further engage children and adolescents, short face‐to‐face interviews (incorporating writing or drawings) were undertaken at the Queensland Children's Hospital in person during routine clinic visits with young patients and their siblings. These interviews were undertaken by an experienced researcher (GD), trained in qualitative data collection with children. We asked question prompts such as: ‘*What matters to you most?’, ‘Is there anything that you feel confused about or wonder about?’, ‘Is there something you're not sure about, like how people get cancer or how doctors help them get better?’, ‘What matters most to you when you think about your condition?’, and ‘Is there anything you think could be done to help kids feel better while getting treatments?*’, inviting them to write or draw their thoughts on any aspect of cancer experience they felt was important and unanswered. All responses from these face‐to‐face interviews were included in the overall dataset and analysed alongside online survey submissions during Phase 3.

### 2.5 Phase 3: Refining Uncertainties to Synthesis Summary Questions for Research

We conducted a qualitative content analysis of submissions. Three authors (GD, NB, and SO) independently reviewed the entries multiple times and coded them using keywords aligned with the PSP scope. We identified potential out‐of‐scope questions and listed them separately. The SG subsequently reviewed and verified these exclusions. We analysed each submission individually and grouped similar entries into seven categories: (1) causes of cancer and prevention; (2) communication and information sharing; (3) treatment and side effects management; (4) service delivery; (5) psychosocial well‐being and support; (6) long‐term effects and survivorship; (7) palliative care, end‐of‐life care and bereavement. Within categories, we mapped similar submissions and combined them into broad summary questions, preserving respondent intent. The summary questions were restructured into a consistent format that involved identifying the target population, specifying the intervention or exposure, and defining the expected outcomes to ensure clarity and consistency for evidence synthesis. We held 18 online subcommittee meetings with selected and volunteered SG members to review, refine and confirm consensus on groupings and wording of each summary question, drawing on their diverse expertise. The decisions from each meeting were then shared with the full SG, who reviewed and agreed on the clustering and the wording of the complete list of synthesised summary questions.

### 2.6 Phase 4: Evidence Verification

We conducted literature reviews to confirm that each summary question remained unanswered by existing evidence. GD developed the search strategies, approved by the SG, and executed with support from a health librarian between January and March 2025. To ensure the reviews are relevant and feasible, within the scope of a rapidly evolving, updated evidence‐rich field, we limited our searches to evidence published in the last ten years, focusing on studies that synthesised existing research, such as systematic reviews and qualitative meta‐syntheses or large, nationally representative cohort studies. While this approach aligns with JLA guidance to adopt proportionate and pragmatic evidence‐checking methods, it carries the inherent risk of overlooking older but still valid studies, particularly in areas where research progresses more slowly or where long‐term follow‐up evidence spans extended periods. We mitigated this by consulting national guidelines and trial databases, identifying foundational studies when uncertainty existed, and seeking expert input from the Steering Group before classifying a question as a true uncertainty. GD and SO screened all potentially relevant evidence, with second opinions sought from SG according to their areas of expertise, providing the final consensus on its uncertainty status. Summary questions that were only partially or not answered at all were classified as true uncertainties and included in the interim prioritisation survey. Further details on evidence verification are included in Appendix A.

### 2.7 Phase 5: Interim Prioritisation

We prepared the online interim prioritisation survey (Survey 2) in Qualtrics, launched and kept it live for 2 months. Survey 2 presented the summary questions within the seven categories, with the order of categories and order of summary questions randomised for each participant to minimise bias and fatigue. Eligibility, recruitment, and promotion mirrored Survey 1, with additional direct invitations sent to Survey 1 respondents who had provided contact details for future participation. We invited respondents to create a short‐list by selecting any number of the summary questions they considered important and then to refine their choices to a final selection of up to 10 summary questions. Following JLA PSP methodology, we used a consensus‐focused rather than inferential, frequency‐based scoring approach to rank the longlisted summary questions, ensuring that priorities reflect stakeholder input without introducing assumptions about underlying distributions or effect sizes of the responses. Responses were analysed by GD descriptively across three groups: patients/survivors, caregivers, and health professionals, to ensure equal weighting of perspectives regardless of differences in group size. For each summary question, we tallied the number of votes from respondents in each group. We then ranked the summary questions by frequency of votes, assigning a rank of 1 to the question with the most votes, equating to a score of 1. Questions with the lowest scores represented the highest priorities, as they received the most votes. We compared these rankings across groups and subgroups (based on age, sex, cultural representation and the cancer type) to explore similarities and differences in priorities. These group comparisons maintained the stability of top‐ranked questions across demographic subsets throughout the analytical approach and were not dependent on specific scoring assumptions or stakeholder representation. This further demonstrates that the highest‐ranked priorities were stable across diverse demographic subsets and were not dependent on scoring assumptions or group size differences, while ensuring that the lower‐ranked questions were not taken forward. The SG reviewed the rankings and confirmed the top‐ranked summary questions to take forward to final prioritisation.

### 2.8 Phase 6: Finalising the Top Ten Research Priorities

We opened our expression of interest for participation in the final workshop at the same time that Survey 2 was launched, and widely shared this opportunity via relevant networks and social media. Given the number of participants was limited for the workshop, the SG purposefully selected participants from among the expressions of interest, aiming to achieve diversity across stakeholder groups, age, gender, professional role, and geographical location. GD contacted the selected individuals to confirm their participation and discuss any accessibility requirements. Participants with lived experience were remunerated for workshop participation in line with the Remuneration Guide for Consumer Involvement in Health Research [32].

We conducted a full‐day online workshop via Zoom on 27th June 2025. Before the workshop, participants were sent the top‐ranked summary questions and relevant preparation materials. Participants were requested to rank their top 10 or select their top and bottom three. The workshop was chaired by SK, supported by two additional independent JLA Facilitators. The workshop involved two sessions of small group discussion, with breaks in between. During session 1, participants were divided into three groups, each with a mix of individuals with lived experience and health professionals to ensure a balance of professionals from different disciplines, survivors and parents. Each group was assigned a JLA Facilitator to coordinate the discussion and an observer, who was not involved in decision‐making during the process. During session 1, each participant was asked to share the three summary questions they ranked highest and lowest in their individual ranking. The Facilitators arranged the questions according to the participant rankings, into three areas on each group's screen: higher priority, medium priority and lower priority. During the latter half of session 1, groups further discussed and re‐ordered the priorities to establish three lists with initial rankings. At the end of session 1, the JLA Facilitators combined the rankings across each group into an overall ranking. These were calculated by taking the average rank across the three groups for each summary question, yielding an overall ranking for discussion. The summary question with the lowest score was ranked #1, i.e., the highest priority, and the highest score was ranked lowest. When two or more scores were equal, the geometric mean was used to confirm the rank order. In session 2, participants were reallocated into new groups. They further discussed and revised the combined ranked list, clarifying and comparing the reasons for their rank of priorities in each group. Participants were encouraged to share their views and experiences and to consider other people's opinions throughout. Through facilitated discussion, the small groups re‐ordered the questions from highest to lowest priority. The final rankings across each small group were again combined and identified the overall final ranking for each summary question, following the same approach as in session 1. This resulted in the final top 10 research priorities. Finally, all participants reconvened in a whole group plenary session to reflect on the Top 10 priorities.

## 3 Results

### 3.1 Collecting Uncertainties

A total of 229 people submitted 701 uncertainties in Survey 1, with an average of three research questions per respondent. Of these, 99 submissions were deemed outside the agreed scope of the PSP and were unanswerable by research and removed from the analysis. The largest group of respondents were caregivers (*n* = 116, 51%), followed by professionals (*n* = 68, 30%) and patients/survivors (*n* = 41, 19%). The patients/survivors’ cohort included young patients and survivors (≤ 18 years) and adult survivors of child and adolescent cancer (> 18 years). Four (2%) respondents reported mixed experience, either as caregivers and health/social professionals or survivors and health/social professionals. Most respondents identified as female (*n* = 195, 85%), and the largest proportion of respondents was from Queensland (*n* = 84, 37%). Aboriginal and Torres Strait Islander peoples were represented by 12 (4.6%) participants, aligning with the 2021 census [33] and literature which reported 3.8% of the Australian population identified as Aboriginal and Torres Strait Islanders [34]. Haematological malignancies accounted for the largest proportion of respondents among both patients/survivors (*n* = 21, 49%) and caregivers 41% (*n* = 48, 41%). Further details on demographics are included in Table 1.

**Table 1:** Demographics of participants across each priority setting partnership stage.

	Survey 1 *n* = 229 (%)	Survey 2 *n* = 474 (%)	Workshop *n* = 27 (%)
**Participant category**			
** Health/social care professional**	**68 (29%)**	**139 (29%)**	**11 (41%)**
Medical doctors	13 (19%)	29 (%)	3 (%)
Nurses	16 (24%)	31 (%)	2 (%)
Allied professionals (pathologists, physiotherapists, radiotherapists, dietitians, clinical psychologists, occupational therapists, genetic counsellors, pharmacists, art/music therapists, social workers)	40 (58%)	79 (57%)	6 (22%)
** Caregivers**	**116 (51%)**	**280 (59%)**	**9 (33%)**
Parents	93 (80%)	239 (85%)	9 (100%)
Relatives/kinship	8 (7%)	23 (9%)	0
Siblings	15 (13%)	18 (6%)	0
Siblings ≤ 18 years	9 (60%)	0	0
Siblings > 18 years	6 (40%)	18 (100%)	0
** Patients/survivors**	**41 (18%)**	**55 (12%)**	**7 (26%)**
Patients ≤ 18 years (on treatments)	20 (49%)	12 (22%)	0
Survivors > 18 years (Finished treatment in the last 12 months or more)	21 (51%)	43 (78%)	7
** Mixed**	**4 (1.7%)**	**0**	**0**
**Gender**			
Woman or female	184 (80%)	358 (76%)	20 (74%)
Man or male	32 (14%)	82 (17%)	7 (26%)
Non‐binary	2 (1%)	10 (2%)	0
Not answered	11 (5%)	24 (5%)	0
**Age**			
≤ 18 years	29 (13%)	12 (3%)	0
≤ 10 years	2 (7%)	2 (17%)	0
11–14 years	21 (72%)	8 (67%)	0
15–18 years	6 (21%)	2 (17%)	0
18–24 years	16 (7%)	49 (11%)	5 (19%)
25–34 years	23 (10%)	68 (14%)	1 (4%)
35–44 years	68 (29%)	149 (31%)	5 (19%)
45–54 years	56 (24%)	118 (25%)	15 (54%)
55–64 years	25 (11%)	48 (10%)	1 (4%)
65 or more years	4 (2%)	15 (3%)	0
Not answered	8 (4%)	15 (3%)	0
**State**			
Queensland	84 (37%)	131 (28%)	10 (36%)
New South Wales	39 (17%)	115 (24%)	7 (26%)
Northern Territory	4 (2%)	9 (2%)	0
South Australia	16 (7%)	51 (10%)	1 (4%)
Tasmania	7 (3%)	11 (3%)	1 (4%)
Victoria	51 (22%)	92 (19%)	7 (26%)
Western Australia	14 (6%)	37 (8%)	1 (4%)
Not answered	14 (6%)	28 (6%)	0
**Australian Aboriginal and/or Torres Strait Islander**			
Yes	12 (5%)	39 (8%)	1 (4%)
No	164 (71%)	323 (68%)	26 (96%)
I am not sure/I don't know	3 (2%)	1 (0.2%)	0
Prefer not to say	9 (5%)	57 (12%)	0
Not answered	41 (17%)	54 (11.8%)	0
**Cancer type** a	**(161** a **)**	**(335** a **)**	N/A
Blood cancer (e.g., leukaemia or lymphoma)	69 (43%)	163 (49%)	N/A
Brain or spinal tumour	26 (16%)	55 (16%)	N/A
Solid tumour (e.g., neuroblastoma, bone tumour)	46 (29%)	89 (27%)	N/A
Not answered	19 (12%)	28 (8%)	N/A

Abbreviation: N/A, not available.

^a^ Only patients/survivors and caregivers respond to this question.

**Figure 2:**
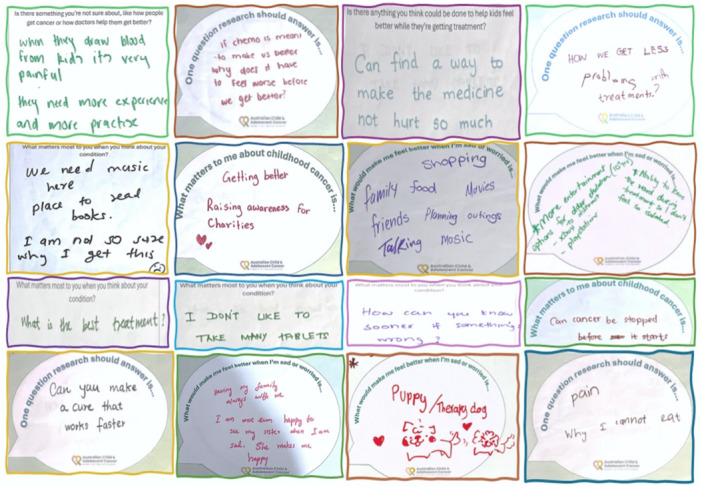
Examples of responses received from young people included in the PSP.

We received 49 in‐scope submissions from 29 (13%) respondents under 18 years; 20 child and adolescent cancer patients and nine siblings. Children at the age of 11–14 years predominated (*n* = 21, 72%) in Survey 1, with fewer older adolescents (15–18 years) (*n* = 6, 21%) and only two children ≤ 10 years (7%). A few examples of submissions collected at interviews at the Queensland Children's Hospital are illustrated in Figure 2.

### 3.2 Refining Uncertainties to Synthesise Summary Questions for Research

We categorised 602 in‐scope submissions into seven categories, with some representing more than one category. Following JLA guidance, subcommittees consisting of survivors, caregivers, and health professionals, from the members of the SG, independently reviewed all submissions within each category. An initial set of 93 summary questions was created by grouping similar submissions and drafting overarching questions that capture closely related submissions. Subcommittee members independently highlighted draft questions that addressed similar concepts, populations, or intervention areas. Similar questions were merged and subsequently refined into broader one question during subcommittee meetings. Some questions were split when a single draft question encompassed multiple distinct submissions not appropriately captured by one item. Each proposed merge or split was discussed using a consensus approach. Submissions were rechecked against the refined questions to ensure that each summary question accurately represented the underlying submissions and to check for duplication or misclassification. Wording was revised to ensure readability, neutrality, and alignment with the scope. This process resulted in a final set of 49 summary questions, distributed across seven categories, as illustrated in Table 2.

**Table 2:** Distribution of summary questions within each category.

Themes	Number of individual submissions	Number of summary questions synthesised
Causes of cancer and prevention	44	5
Communication and information sharing	76	5
Long‐term effects and survivorship	108	6
Psycho‐social well‐being and support	148	10
Palliative care, end‐of‐life care and bereavement	15	6
Service delivery	99	10
Treatment and side effects management	139	7
Total (in‐scope)	**602**	**49**
**Out‐of‐scope submissions**	**99**	

### 3.3 Evidence Verification

Of the 49 summary questions, we determined that 39 (80%) were unanswered, with 10 (20%) partially answered. The questions that were classified as partially answered had some existing relevant evidence but lacked sufficient information to fully resolve the uncertainties and were therefore kept for prioritisation. As a result, all 49 questions were classified as true uncertainties.

### 3.4 Interim Prioritisation

A total of 474 individuals responded to Survey 2. Survey 2 received more than double the number of responses compared with Survey 1. The majority of the respondents were caregivers (n = 280, 59%), comprising largely of parents (n = 245, 88%). This was followed by 139 (29%) professionals and 55 (12%) patients/survivors. Further details on the Survey 2 demographics are included in Table 1.

Reviewing the results across respondent groups (Appendix B), we selected the top 7 summary questions from each group of patients/survivors, caregivers and healthcare professionals, which produced a consolidated list of 16 summary questions. Subsequently, we examined ranked questions by subgroups, adding three frequently recurring summary questions across subgroups of age category, sex, cancer type and Indigenous status to expand the list to 19. The list of 19 summary questions was taken forward to the final workshop.

### 3.5 Top 10 Prioritisation

We received a total of 83 expressions of interest to participate in the workshop. Of these, we identified 18 to be fraudulent based on factors such as an IP address outside of Australia and disconnected phone numbers. We screened the 65 expressions of interest deemed eligible and purposively selected 30 with consideration for maximum diversity. The workshop included 27 participants representing multiple Australian states, and experiences of child and adolescent cancer: 7 survivors, 9 parents, and 11 health professionals in a wide range of disciplines (Table 1).

During the sessions, many participants initially selected priorities based on their personal or professional experiences; however, structured group discussions prompted shifts in perspective as individuals considered broader viewpoints. Through facilitated workshop discussions and reflection, the stakeholder group participants ranked all 19 summary questions (Appendix C) and reached consensus on the final Top 10 research priorities for child and adolescent cancer in Australia. Early in the workshop discussions, participants expressed the difficulty of differentiating lower‐priority topics, as all 19 questions were viewed as important and warranting further research. While many participants initially approached the task based on their personal or professional experiences, group discussions encouraged broader perspectives and recognition of the challenge of accommodating individual preferences within a collective decision‐making process. Participants also reflected on how broadly versus narrowly defined research questions might be interpreted, noting that the direction of future research based on these priorities would depend on how researchers choose to frame and operationalise these priorities. At the end of the workshop, participants reflected positively on the range of focus areas represented in the final Top 10, which captured a broad range of experiences from diagnosis and treatment to survivorship, end‐of‐life care, and bereavement. The final Top 10 priorities are presented in Table 3. Participants further emphasised the value of making the full list of 49 summary questions and 602 original submissions publicly available to guide future research beyond the Top 10.

**Table 3:** Final top 10 research priorities for child and adolescent cancer in Australia.

Rank	Theme	Research priorities
1	Treatment and side effects management	How can the development of cancer treatments and equitable access to treatment be optimised for children and young people so that treatments are safer, less toxic, less traumatic, and more effective?
2	Long‐term effects & survivorship	What are the best ways to prevent, identify and manage the long‐term effects of cancer and its treatment in children and young people on physical and psychological health?
3	Long‐term effects & survivorship	What are the best ways to support children and young people through transitions from active cancer treatment to follow‐up and everyday life? (e.g., re‐engagement with education and communities and transition to adult healthcare)?
4	Long‐term effects & survivorship	What is the effect of cancer survivorship programs for children and young people on health outcomes and healthcare costs?
5	Psycho‐social well‐being & support	How can psychosocial interventions that prevent or minimise the trauma of cancer treatment in children and young people be better integrated to care?
6	Service delivery	How can health and community services better engage with First Nations children and young people with cancer, their kin and families to provide culturally safe care and support services?
7	Treatment and side effects management	How can a more personalised and tailored approach to childhood cancer treatment be developed and delivered to meet the unique needs of each person?
8	Service delivery	How can children and young people with cancer from regional, rural and remote locations of Australia, and their families, access better support services? (e.g. when travelling for treatment, living away from home or seeking services)
9	Psycho‐social well‐being & support	What are the psychosocial effects of cancer in children and young people, and their families, during and after treatment, and how can these effects be addressed?
10	Treatment and side effects management	What factors contribute to the risk of cancer relapse or treatment resistance in children and young people? Can identifying these factors early help personalise treatment to improve outcomes?

### 3.6 Dissemination

The final research priorities were officially launched at the Australia and New Zealand Children's Haematology and Oncology Group (ANZCHOG) Annual Scientific Meeting in 2025 and disseminated to both the targeted and broader research communities. In addition to formal channels, the Top 10 priorities were promoted via social media to increase visibility and engagement. We are currently working with partner organisations and relevant professional bodies to support their uptake and implementation of these priorities. A comprehensive report detailing the outcomes is currently in preparation. Upon completion, we will share the report with key research institutions, national funding agencies, and philanthropic bodies to further promote the priorities and stimulate targeted funding calls that address these critical areas. It ensures that the unique needs of children, young people, and families are fully embedded within national cancer policy and practice.

## 4 Discussion

This PSP brought together children, adolescents, families, and health professionals to identify the top 10 research priorities for child and adolescent cancer in Australia. Survey 1 revealed that research uncertainties grounded in lived experience spanned the entire disease trajectory, from aetiology, diagnosis, through survivorship and beyond. Our process filtered these uncertainties, and the final priorities were from four categories: (1) treatment and side effects management; (2) long‐term effects and survivorship; (3) psycho‐social wellbeing and support; and (4) service delivery. These categories broadly represent the multifaceted short and long‐term challenges faced by the child and adolescent cancer community.

Three of Australia's top child and adolescent cancer research priorities identified through this PSP highlight critical gaps in current knowledge and practice regarding cancer treatment safety, toxicity, and resistance. Despite rising national childhood cancer cure rates, the PSP emphasised the need for more research on safer, effective therapies (Priority #1) and relapse and treatment resistance (Priority #10), adding personalised approaches (Priority #7). Progress in drug development is slowed by disease heterogeneity, rarity, limited paediatric‐specific evidence, and constraints on large‐scale trials [35]. The scarcity of paediatric‐specific targeted therapies, driven by barriers to exploring therapeutic potential in small populations, inconsistent outcome reporting, and limited evaluation of precision medicine, further hinders advancement [36]. While strategies such as immunotherapy and precision medicine offer progress, they demand rigorous evaluation and sustained investment to ensure safety and efficacy, as highlighted in the National Framework for Genomics in Cancer Control [37].

As survival rates improve, long‐term effects have become increasingly prominent, often emerging years post‐treatment and impacting quality of life [3]. Although survivorship programs are expanding across Australia, evidence remains limited regarding risk prediction and optimal interventions to prevent or mitigate late effects, along with inequities in access and outcomes, particularly in rural and remote areas [38]. Priorities #2 and #4 emphasise the need for coordinated, evidence‐informed survivorship care, supported by robust outcome and economic evaluations. Priority #3 underscores the challenges of transition from treatment to survivorship, including shifts to adult healthcare and reintegration into school and community life. Addressing these complex medical, psychological, educational, and social needs requires sustained, multidisciplinary approaches [39]. However, effective care models remain unclear and context‐dependent [40, 41], reinforcing the need for tailored research to guide best practice.

Psychosocial challenges in child and adolescent cancer are constantly evolving and shaped by their circumstances, impacting both patients and their families at every stage of the care journey. Further investigation into these needs is identified as essential (Priority #9), emphasising the importance of age‐appropriate psychosocial interventions that target specific psychosocial parameters and can be delivered through diverse modalities to address the unique challenges encountered throughout survivorship [42, 43]. Although such interventions show benefit for patients and families [44, 45], evidence on their integration into routine care remains limited. In particular, trauma‐informed and family‐centred approaches continue to be inconsistently embedded within standard practice (Priority #5). Persistent gaps in effective care models, timing, and delivery, compounded by resource constraints, uneven access to trained professionals, and fragmented systems, continue to hinder consistent support and long‐term outcomes of interventions [46, 47].

Despite advancements, disparities in child and adolescent cancer care persist, disproportionately affecting First Nations children and those in regional, rural, and remote areas in Australia [24, 48]. Priority #6 calls for culturally safe care that honours Indigenous perspectives, while Priority #8 highlights the challenges faced by families who must travel or relocate for treatment. Yet, culturally safe, context‐specific, and Indigenous‐led approaches remain under‐researched, and service delivery is often fragmented and adult‐focused [49]. Achieving equity requires tailored, accessible, and responsive services that address the diverse needs of children and families across Australia [24].

The proposed priorities represent broad research areas rather than discrete questions, within which multiple, specific research questions can be developed, providing a foundation for comprehensive programs to improve outcomes for children, adolescents, and families affected by cancer in Australia. Notably, the top two priorities retained their ranking across both Survey 2 and the final workshop, underscoring their shared relevance among stakeholders. Several other priorities received comparable scores, reflecting broad consensus and affirming the inclusivity of the PSP process. While the finalised top 10 priorities represent the most pressing areas identified within the scope of the partnership, research areas beyond the top 10 remain interrelated with the identified priorities and may provide essential context or a foundational role in advancing priority areas through integrated approaches. Availability of the broader datasets with submitted uncertainties and summary questions [50] offers valuable insights that future prioritisation projects can use to guide funding, identify emerging themes, and support transparent, inclusive decision‐making. Therefore, the prioritisation process should be viewed not as a narrowing of focus, but as a framework that highlights immediate needs while recognising the ongoing value of wider research contributions. Thus, ongoing monitoring and evaluation of the translatability of priorities is essential to ensure that future child and adolescent cancer research remains responsive to community needs, actionable, and embedded within national policy and funding frameworks.

Importantly, we sought to incorporate the perspectives of young patient voices wherever possible. Despite employing tailored communication strategies and child‐friendly formats, meaningful engagement with children and adolescents proved challenging. In survey 1, 13% of patient/survivors who responded were aged under 18 years, falling to just 3% in survey 2, with caregivers substantially outnumbering young voices. This is not unexpected, given that approximately half of children diagnosed and treated for cancer are aged 0–4 years [1], as they are too young to participate. As child cancer survivors grow older, they may advocate for aspects of care they are currently navigating; however, this does not capture the significant portion of their cancer journey that occurred during their early childhood. In this context, parents and carers play an essential role in representing those experiences. Thus, parent representation within this PSP was a necessary complement to the voices of young people. Conversely, reduced participation by younger respondents may also reflect survey fatigue when engaging with lengthy, text‐heavy online surveys or reduced direct clinical recruitment at the interim stage. We supplemented Survey 1 with child‐specific face‐to‐face interviews and added visual supports to Survey 2 to improve accessibility as per the international guidance provided by Postma et al., 2025 [51] and aligned with recent work highlighting the need for age‐appropriate, flexible engagement methods [18]. While children's qualitative contributions were fully integrated into the thematic analysis and summary‐question development, future PSPs may benefit from more child‐tailored methods to increase youth participation in prioritisation phases and ensure that young people's experiences continue to shape research and priorities to inform future research and care strategies. These top priorities align closely with the objectives of the Australian Cancer Plan [52], highlighting innovation in treatment, survivorship, supportive care, and equitable access, particularly for priority populations such as First Nations communities and those in rural and remote areas. The Plan also encourages translational research into broader determinants of health, including social, cultural, commercial, and environmental factors such as early childhood environments, income, food security, housing, and healthcare access. While child‐specific focus areas include genetic causes, risk factors, and strategies to expand genomic testing and risk‐based screening, several critical issues remain underrepresented in national research and the policy landscape. These include treatment‐related toxicity and trauma, relapse and resistance, transitions back to school and community life, and systematic evaluation of paediatric survivorship programs that address the unique developmental, psychological, and social needs of young patients and their families. Notably, the top‐ranked Australian priority converges with the number one priority identified by the UK Children's Cancer PSP [19]. Both emphasise the urgent need for treatments that are more effective, safer, and less toxic for children and young people. This alignment highlights a shared international concern regarding treatment‐related harms and underscores that improving survivorship quality is as critical as improving survival. The consistency across two independently conducted PSPs suggests that developing more tolerable and equitable treatments is a global research imperative, reinforcing the value of cross‐country collaboration and coordinated research investment.

### 4.1 Strengths and Limitations

We utilised the established JLA methodology, a rigorous process for identifying research priorities that has been successfully implemented across diverse health conditions, populations, and settings. This approach enabled meaningful collaboration among individuals diagnosed with cancer, their families, and health and social professionals; groups often underrepresented in research decision‐making, ensuring that the final priorities reflected lived experience and frontline clinical concerns.

Methodological and administrative constraints, including ethics approvals, governance requirements, and logistical complexities, restricted recruitment efforts, with data collection limited to the hospital affiliated with the principal investigator's institution. Children were not included in the final prioritisation workshop due to feasibility considerations associated with conducting a full‐day consensus‐based workshop in an online format. Concerns included consent, comprehension of complex medical terminology, the sensitivity of discussions, and challenges associated with sustained participation alongside adults, as well as missing school. While children were therefore not directly involved in the final prioritisation workshop, their perspectives were incorporated earlier in the PSP through the survey, enabling them to contribute in an accessible and age‐appropriate manner. The absence of direct child participation in the final consensus phase is acknowledged as a limitation and highlights the need for future priority‐setting initiatives to adopt developmentally appropriate engagement methods.

Having used similar promotional approaches, we have noted a significant difference in the demographic composition and volume of respondents between Survey 1 and Survey 2. The higher participation of caregivers and health professionals in Survey 2 may reflect both reduced response burden with greater feasibility and wider dissemination through stakeholder networks. Certain other groups were also underrepresented in the process, notably medical doctors (19% of health professionals) compared with allied health (58%) and nurses (24%), necessitating reflexive consideration of how respondent composition may have shaped the priorities. Men were also underrepresented across the overall sample, consistent with gendered trends in caregiving, health professions, and research participation [53]. However, we did not use specific data collection approaches during Survey 2 as we did during Survey 1, which was that we held face‐to‐face interviews to recruit young participants. While these variations represent a limitation, the application of comparison in rankings across the stakeholder groups, in line with JLA methodology, helped minimise potential imbalance in the prioritisation outcomes. ensuring that changes in respondent volume did not skew the prioritisation results, and that thematic consistency across stakeholder groups remained central to determining which questions progressed to the final workshop. We acknowledge that these imbalances may have influenced the articulation of research priorities, as these specific population groups may hold distinct perspectives and informational needs.

### 4.2 Implications

This PSP aimed to prioritise real‐world needs and guide future research and equitable, community‐responsive resource allocation, including funding. These priorities reflect unmet needs and offer directions for researchers seeking to foster meaningful patient and public engagement. They highlight the value of co‐developing research with the childhood cancer community and the need for transparent, inclusive communication strategies tailored to diverse audiences. By embedding partnership throughout the research lifecycle, from design to dissemination and through feedback loops, future studies can better align with lived experiences and drive more impactful, patient‐centred outcomes. Although the establishment of these research priorities may not yield immediate changes for children and adolescents and their families affected by cancer, they provide a strategic framework for future research and policy development, ensuring future efforts remain contextually relevant and responsive to patients, families, and communities. Advancing work in these priority areas will also strengthen clinical practice by building a robust evidence base and informing credible clinical guidelines.

## 5 Conclusion

We conducted a James Lind Alliance Priority Setting Partnership to determine Australia's top 10 research priorities for child and adolescent cancer. The breadth of identified priorities, from treatment, survivorship, psychosocial, and health service access and equity, provides a clear, community‐driven roadmap. Aligning future research with the lived experiences of those affected by child and adolescent cancer and clinical experiences will ensure that future efforts are grounded in real‐world needs and have the potential to drive meaningful improvements in care and outcomes. Addressing these priorities is a shared responsibility across funders, researchers, and community organisations.

## Author Contributions

**Gayani De Silva:** conceptualisation, methodology, investigation, data curation, project administration, visualisation, writing – original draft, writing – review and editing, formal analysis, validation. **Eden G. Robertson:** methodology, supervision, formal analysis, writing – review and editing, investigation. **Alison Bowers:** methodology, supervision, writing – review and editing, formal analysis, investigation. **Clarissa Schilstra:** methodology, formal analysis, writing – review and editing, investigation. **Sheila K. Patel:** methodology, formal analysis, writing – review and editing, investigation. **Maria C. McCarthy:** methodology, formal analysis, writing – review and editing, investigation. **Jordana McLoone:** formal analysis, writing – review and editing, investigation, methodology. **Jason D. Pole:** formal analysis, writing – review and editing, methodology, investigation. **Ursula M. Sansom‐Daly:** methodology, formal analysis, writing – review and editing, investigation. **Sabina Oppelaar:** formal analysis. **Joanne Cummings:** methodology, supervision, writing – review and editing, formal analysis. **Natalie Bradford:** methodology, data curation, investigation, formal analysis, supervision, funding acquisition, writing – review and editing, conceptualisation, validation.

## Ethics Statement

This study involves human participants. We received ethics approval from the Children's Hospitals Network Human Research Ethics Committee (HREC/2024/QCHQ/104116). We obtained governance approval to collect data from patients and families at Queensland Children's Hospital from Queensland University of Technology – Health Translation Queensland, and Children's Hospital Queensland and secured administrative approval from Queensland University of Technology to carry out the study as part of a PhD project.

## Conflicts of Interest

The authors declare no conflicts of interest.

## Supporting information

Supporting File

## Data Availability

The data that support the findings of this study are available from the corresponding author upon reasonable request. The majority of data that support the findings of this study are openly available via the James Lind Alliance website at ChildandAdolescentCancer(Australia)|NIHRJLA.

## References

[1] AIWH . Australian Institute of Health and Welfare ‐ Cancer data in Australia ‐ Cancer incidence by age visualisation 2024 [Available from: https://www.aihw.gov.au/reports/cancer/cancer-data-in-australia/contents/cancer-incidence-by-age-visualisation.

[2] AIHW . Australian Institute of Health and Welfare (AIHW), 2024, Cancer data in Australia: Cancer mortality by age visualisation. Canberra: https://www.aihw.gov.au/reports/cancer/cancer-data-in-australia/contents/cancer-mortality-by-age-visualisation [cited September 2025 September 2025].

[3] J C . Many survivors of childhood cancer experience lifelong chronic health problems and shorter lifespans than their healthier peers. https://ascopost.com/news/october-2023/many-survivors-of-childhood-cancer-experience-lifelong-chronic-health-problems-and-shorter-lifespans-than-their-healthier-peers/. The ASCO Post.; 2023 [cited September 2025.

[4] National Health and Medical Research . Strategy Issues Paper – April 2025; Department of Health, Disability and Aging [Report]. Australian Governmnent; 2025 [updated 2 May 2025. Available from: https://www.health.gov.au/resources/publications/national-health-and-medical-research-strategy-issues-paper-april-2025.

[5] A. Fabbri , A. Lai , Q. Grundy , and L. A. Bero , “The Influence of Industry Sponsorship on the Research Agenda: A Scoping Review,” American Journal of Public Health 108, no. 11 (2018): e9–e16. 10.2105/AJPH.2018.304677 PMC6187765 30252531

[6] National Health and Medical Research Council . Community research priorities portal. 2024. https://www.nhmrc.gov.au/research-policy/research-priorities/community-research-priorities-portal2025.

[7] S. Crowe , M. Fenton , M. Hall , K. Cowan , and I. Chalmers , “Patients’, Clinicians’ and the Research Communities’ Priorities for Treatment Research: There Is An Important Mismatch,” Research Involvement and Engagement 1, no. 1 (2015): 2. 29062491 10.1186/s40900-015-0003-x PMC5598091

[8] M. Levelink , M. Voigt‐Barbarowicz , and A. L. Brütt , “Priorities of Patients, Caregivers and Health‐Care Professionals for Health Research – A Systematic Review,” Health Expectations 23, no. 5 (2020): 992–1006. 32643854 10.1111/hex.13090 PMC7696132

[9] J. A. Asamani , S. A. Alugsi , H. Ismaila , and J. Nabyonga‐Orem , “Balancing Equity and Efficiency in the Allocation of Health Resources‐Where Is the Middle Ground?,” Healthcare 9, no. 10 (2021): 1257. 34682937 10.3390/healthcare9101257 PMC8536061

[10] I. Chalmers , M. B. Bracken , B. Djulbegovic , et al., “How to Increase Value and Reduce Waste When Research Priorities Are Set,” Lancet 383, no. 9912 (2014): 156–165. 24411644 10.1016/S0140-6736(13)62229-1

[11] J. D. Harrison , A. D. Auerbach , W. Anderson , et al., “Patient Stakeholder Engagement in Research: A Narrative Review to Describe Foundational Principles and Best Practice Activities,” Health Expectations 22, no. 3 (2019): 307–316. 30761699 10.1111/hex.12873 PMC6543160

[12] L. Esmail , E. Moore , and A. Rein , “Evaluating Patient and Stakeholder Engagement in Research: Moving From Theory to Practice,” Journal of Comparative Effectiveness Research 4, no. 2 (2015): 133–145. 25825842 10.2217/cer.14.79

[13] R. Al‐Shahi Salman , E. Beller , J. Kagan , et al., “Increasing Value and Reducing Waste in Biomedical Research Regulation and Management,” Lancet 383, no. 9912 (2014): 176–185. 24411646 10.1016/S0140-6736(13)62297-7 PMC3952153

[14] JLA. James Lind Alliance . James Lind Alliance priority setting partnerships, 2025 [Accessed date 2 October 2025]. http://www.jla.nihr.ac.uk/.

[15] WHO . *Research and development landscape for childhood cancer: a 2023 perspective*, 2023.

[16] K. M. Primm , E. Blackman , B. M. Sansbury , S. K. Zaidi , and R. Sengupta , “Aacr Pediatric Cancer Progress Report 2025,” Clinical Cancer Research 32, no. 3 (2026): 465–467. 41635220 10.1158/1078-0432.CCR-25-4722

[17] S. Aldiss , L. A. Fern , R. S. Phillips , et al. Research Priorities for Young People With Cancer: A UK Priority Setting Partnership With the James Lind Alliance. *BMJ Open* 9, no. 8 (2019): e028119. http://europepmc.org/abstract/MED/31383701. 10.1136/bmjopen-2018-028119 PMC6688702 31383701

[18] S. Aldiss , P. Hart‐Spencer , L. Langton , et al., “What Matters to You? Engaging With Children in the James Lind Alliance Children's Cancer Priority Setting Partnership,” Research Involvement and Engagement 9, no. 1 (2023): 110. 38037183 10.1186/s40900-023-00518-2 PMC10688066

[19] S. Aldiss , R. Hollis , B. Phillips , et al., “Research Priorities for Children's Cancer: A James Lind Alliance Priority Setting Partnership in the UK,” BMJ Open 13, no. 12 (2023): e077387. 10.1136/bmjopen-2023-077387 PMC11148658 38128939

[20] JLA . Pediatric Cancer (Canada) PSP Top 10 Research Priorities 2021. https://www.jla.nihr.ac.uk/documents/pediatric-cancer-canada-psp-protocol.

[21] Adolescent and Young Adult Cancer (Canada), 2024. https://www.jla.nihr.ac.uk/priority-setting-partnerships/adolescent-and-young-adult-cancer-canada#tab-28891.

[22] K. Milley , P. Druce , M. McNamara , et al., “Cancer in General Practice Research Priorities in Australia,” Australian Journal of General Practice 53 (2024): 227–234. 38575544 10.31128/AJGP-02-23-6699

[23] F. Crawford‐Williams , B. Koczwara , R. J. Chan , et al., “Defining Research and Infrastructure Priorities for Cancer Survivorship in Australia: A Modified Delphi Study,” Supportive Care in Cancer 30, no. 5 (2022): 3805–3815. 35031828 10.1007/s00520-021-06744-2

[24] A. Truong , K. Williams‐Tucker Ngarluma Wongutha Wudjari Noongar , A. Narkle Whadjuk Goreng Noongar , et al., “Current Gaps in Knowledge and Future Research Directions for Aboriginal and Torres Strait Islander Children With Cancer,” Medical Journal of Australia 222, no. 10 (2025): 524–528. 40207417 10.5694/mja2.52650 PMC12126966

[25] S. Medlow and P. Patterson , “Determining Research Priorities for Adolescent and Young Adult Cancer in Australia,” European Journal of Cancer Care 24, no. 4 (2015): 590–599. 25684198 10.1111/ecc.12291

[26] C. E. Schilstra , U. M. Sansom‐Daly , M. Schaffer , et al., “‘We Have All This Knowledge to Give, So Use Us as a Resource’: Partnering With Adolescent and Young Adult Cancer Survivors to Determine Consumer‐Led Research Priorities,” Journal of Adolescent and Young Adult Oncology 11 (2022): 211–222. 34297633 10.1089/jayao.2021.0052

[27] T. Clinton‐McHarg , C. Paul , R. Sanson‐Fisher , C. D'Este , and A. Williamson , “Determining Research Priorities for Young People With Haematological Cancer: A Value‐Weighting Approach,” European Journal of Cancer 46, no. 18 (2010): 3263–3270. 20634057 10.1016/j.ejca.2010.06.013

[28] JLA . The James Lind Alliance Guidebook, 2021.

[29] Protocol:. Australian Child and Adolescent . Cancer PSP JLA website: NIHR JLA; 2024 [Available from: https://www.jla.nihr.ac.uk/documents/australian-child-and-adolescent-cancer-psp-protocol.

[30] A. Tong , A. Synnot , S. Crowe , et al., “Reporting Guideline for Priority Setting of Health Research (Reprise),” BMC Medical Research Methodology 19, no. 1 (2019): 243. 31883517 10.1186/s12874-019-0889-3 PMC6935471

[31] ACACanPSP . Australian child and adolescent cancer research PSP 2024 [Available from: https://www.auschildcanpsp.org/.

[32] Position statement ‐ Remuneration and reimbursement of consumers: Health Consumers Queensland [Available from: https://www.hcq.org.au]

[33] ABS . Australian Bureau of Statistics, Aboriginal and Torres Strait Islander people: Census, 2021. [updated 2022. Available from: https://www.abs.gov.au/census/find-census-data/quickstats/2021/IQSAUS.

[34] D. R. Youlden , P. D. Baade , C. A. McBride , et al., “Changes in Cancer Incidence and Survival Among Aboriginal and Torres Strait Islander Children in Australia, 1997‐2016,” Pediatric Blood & Cancer 69, no. 4 (2022): e29492. 34908222 10.1002/pbc.29492

[35] P. J. Houghton and R. T. Kurmasheva , “Challenges and Opportunities for Childhood Cancer Drug Development,” Pharmacological Reviews 71, no. 4 (2019): 671–697. 31558580 10.1124/pr.118.016972 PMC6768308

[36] J. Lee , L. Gillam , K. Visvanathan , J. R. Hansford , and M. C. McCarthy , “Clinical Utility of Precision Medicine in Pediatric Oncology: A Systematic Review,” JCO Precision Oncology 5, no. 5 (2021): 1088–1102. 34994630 10.1200/PO.20.00405

[37] National Framework for Genomics in Cancer Control Cancer Australia , 2025. https://www.canceraustralia.gov.au/key-initiatives/national-framework-genomics-cancer-control.

[38] R. E. Hill , J. E. Fardell , R. Mercieca‐Bebber , et al., “Survivorship Care Plan Utilization in Australia and New Zealand: Survivors’, Parents’ and Healthcare Providers’ Perspectives,” Supportive Care in Cancer 33, no. 3 (2025): 182. 39937313 10.1007/s00520-025-09238-7 PMC11821783

[39] M. Conway Keller , C. King , L. Hart , et al., “The End of Cancer Treatment Experience for Children, Adolescents, and Their Parents: A Systematic Review of the Literature,” Journal of Psychosocial Oncology 38, no. 5 (2020): 573–591. 32602790 10.1080/07347332.2020.1769795

[40] C. Signorelli , C. E. Wakefield , J. K. McLoone , et al., “Models of Childhood Cancer Survivorship Care in Australia and New Zealand: Strengths and Challenges,” Asia‐Pacific Journal of Clinical Oncology 13, no. 6 (2017): 407–415. 28670761 10.1111/ajco.12700

[41] N. Bradford , R. J. Chan , X. Skrabal Ross , et al., “Childhood Cancer Models of Survivorship Care: A Scoping Review of Elements of Care and Reported Outcomes,” Journal of Cancer Survivorship 19, no. 6 (2025): 1995–2011. 38722536 10.1007/s11764-024-01610-6 PMC12546495

[42] N. O'Donnell , L. Ellis , J. E. Morgan , et al., “Psychosocial Interventions to Improve Wellbeing in Teenage and Young Adult Post‐Treatment Survivors of Childhood Cancer: A Systematic Review,” Psycho‐Oncology 34, no. 2 (2025): e70081. 39921358 10.1002/pon.70081 PMC11806280

[43] T. Arpaci and N. Altay , “Psychosocial Interventions for Childhood Cancer Survivors: Systematic Review and Meta‐Analysis of Randomized Control Trials,” European Journal of Oncology Nursing 69 (2024): 102541. 38460392 10.1016/j.ejon.2024.102541

[44] I. J. Eche , M. Yusufov , D. A. Isibor , and J. Wolfe , “A Systematic Review and Meta‐Analytic Evaluation of Psychosocial Interventions in Parents of Children With Cancer With An Exploratory Focus on Minority Outcomes,” Pediatric Blood & Cancer 68, no. 12 (2021): e29328. 34523798 10.1002/pbc.29328

[45] A. Coughtrey , A. Millington , S. Bennett , et al., “The Effectiveness of Psychosocial Interventions for Psychological Outcomes in Pediatric Oncology: A Systematic Review,” Journal of Pain and Symptom Management 55, no. 3 (2018): 1004–1017. 28962919 10.1016/j.jpainsymman.2017.09.022

[46] U. M. Sansom‐Daly , J. K. McLoone , J. E. Fardell , et al., “Bridging the Gap: Embedding Psychosocial Oncology Research into Comprehensive Cancer Care for Children and Young People,” Cancers 17, no. 13 (2025): 2123. 40647422 10.3390/cancers17132123 PMC12248999

[47] V. Paul , L. Inhestern , D. Sigmund , et al., “Addressing Gaps and Enhancing Experiences in Support Services for Families of Pediatric Cancer Survivors,” Pediatric Research 98, no. 1 (2025): 168–173. 38886508 10.1038/s41390-024-03320-2 PMC12411228

[48] S. Jessop , S. Hill , K. Bicanin , et al., “Aboriginal Children With Cancer: The Patient and Healthcare Worker Perspective,” Pediatric Blood & Cancer 71, no. 1 (2024): e30747. 37880841 10.1002/pbc.30747

[49] S. Bay , E. V. Taylor , M. Robinson , L. Pilkington , and S. C. Thompson , “Aboriginal and Torres Strait Islander Children and Cancer: A Narrative Review of Incidence, Mortality, Barriers to Diagnosis and Treatment, Psychosocial Needs and Interventions,” The Lancet Regional Health ‐ Western Pacific – Western Pacific 61 (2025): 101530. 40922817 10.1016/j.lanwpc.2025.101530 PMC12414359

[50] JLA . Uncertanities Submitted‐ final data set JLA, 2025, https://www.jla.nihr.ac.uk/priority-setting-partnerships/australian-child-and-adolescent-cancer#tab-key-documents.

[51] L. Postma , F. Gibson , J. Z. Jagt , et al., “Exploring Experiences and Designing Guidance for Involving and Engaging Children and Young People in James Lind Alliance Priority Setting Partnerships,” Health Expectations 28, no. 2 (2025): e70195. 40025789 10.1111/hex.70195 PMC11873199

[52] CancerAustralia . Australian Cancer Plan, 2025.

[53] M. Jang and A. Vorderstrasse , “Socioeconomic Status and Racial or Ethnic Differences in Participation: Web‐Based Survey,” JMIR Research Protocols 8, no. 4 (2019): e11865. 30969173 10.2196/11865 PMC6479282

