# Supplementary material for: What Matters Most: The Top 10 Child and Adolescent Cancer Research Priorities in Australia
Source: Health Expect. 2026 May 16;29(3):e70689. doi: 10.1111/hex.70689 (PMC13179753; doi:10.1111/hex.70689)
Supplement: Supplementary file 1 — Supporting File [file HEX-29-e70689-s001.docx]

## **Appendix A: Information in the evidence verification process**

## Search strategy

- systematic review* OR meta analys* OR meta-analys* OR metaanaly* OR meta synthes* OR meta-synthes* OR review* OR guideline*)

**AND**

- child OR adolescent OR pediatric* OR paediatric* OR teen* OR young* OR childhood

**AND**

- cancer OR neoplasm OR oncology OR tumor OR tumour OR malignancy OR "cancer survivors" OR carcinoma*

**AND**

Parameters

- *additional search terms to reflect the concepts/focus of each summary question*
- Searches were limited to evidence published from January 2014 to January 2025.
- Searches will be limited to publication in English in a peer-reviewed journal (or peak body, in the case of guidelines)

## Details of resources searched for the evidence verification

| Systematic reviews | Nationally representative cohort studies | Guidelines | Ongoing studies |
| --- | --- | --- | --- |
| - The Cochrane Database of Systematic Reviews (which had reviewed randomised controlled trials (RCTs), quasi RCTs, cluster RCTs, cohort studies, cross-sectional studies, qualitative literature) - Joanna Briggs Institute of Systematic Reviews and Implementation Reports (cancer collection) | MEDLINE  CINAHL PsycINFO Embase  Scopus  Web of Science  Reference lists of identified papers were hand-searched for any additional reviews meeting the inclusion criteria | - Australian Clinical Practice Guidelines Portal - Human Genetics Society of Australia guidelines - Cancer Council - Clinical Guidelines Network - [Victorian Paediatric Integrated Cancer Service](https://pics.org.au/wp-content/uploads/2019/06/VictorianPaediatricOncologyCarePathways_FINAL_May2019-compressed.pdf) - [Children’s Oncology Group](http://www.survivorshipguidelines.org/) - Australian and New Zealand Clinical Trials Registry (ANZCTR) and clinicaltrials.gov | - Clinical trials databases will be reviewed on registries   *An ongoing study may not mean that uncertainty will be addressed. Steering group members were to discuss ongoing studies and document any decisions made because of the information found.   - Expert communication |

## Categorisation of evidence

We undertook a literature search of each finalised summary question (that was deemed in-scope) and categorised each as being:

1. Answered: Reliable, up-to-date systematic reviews, meta-analyses or clinical guidelines have already been published.
2. Partially answered:

- Relevant, reliable and up-to-date systematic reviews, meta-analyses and evidence-based guidelines, but do not address continuing questions, that is, aspects of the questions that are unresolved, or ongoing research uncertainties connected to the original research question.
- Relevant systematic reviews but not up to date (i.e., published before 2014).
- Current clinical trial, but not exhaustive enough to answer the question

1. Not answered: No relevant systematic reviews or clinical guidelines identified

All questions categorised as ‘2’ or ‘3’ were deemed unanswered and taken to interim priority setting.

## **Appendix B: Interim prioritisation rankings by participant group, and overall**

| **SQ code** | **Summary Questions - 49** | **H** | **P** | **C** |
| --- | --- | --- | --- | --- |
| 1 | What are the best ways to prevent, identify and manage the long-term effects of cancer and its treatment in children and young people on physical and psychological health? | 3 | 2 | 1 |
| 2 | How can the development of cancer treatments and equitable access to treatment be optimised for children and young people so that treatments are safer, less toxic, less traumatic, and more effective? | 1 | 1 | 2 |
| 3 | What are the long-term effects of different cancer diagnoses and different treatments on a child’s cognition, education and social health? | 19 | 4 | 3 |
| 4 | What are the psychosocial effects of cancer in children and young people, and their families, during and after treatment, and how can these effects be addressed? | 15 | 11 | 4 |
| 5 | What are the best ways to support children and young people through transitions from active cancer treatment to follow-up and everyday life? (e.g., re-engagement with education and communities and transition to adult healthcare)? | 12 | 15 | 5 |
| 6 | What are the best ways to provide social and emotional support to family caregivers of children and young people diagnosed with cancer, during and after treatment and into survivorship? | 40 | 24 | 6 |
| 7 | How can the needs of families be best identified and addressed when a child's cancer is at risk of or does relapse?​ | 17 | 33 | 7 |
| 8 | How can psychosocial interventions that prevent or minimise the trauma of cancer treatment in children and young people be better integrated to care? | 6 | 8 | 8 |
| 9 | What strategies can improve the design and accessibility of clinical trials in Australia for children and young people with cancer? | 29 | 42 | 9 |
| 10 | What are the financial effects of cancer in children and young people on families and how can financial support be better tailored to meet needs? | 23 | 39 | 10 |
| 11 | What are the best ways to predict, reduce and manage the short and long-term side effects of cancer treatment in children and young people? | 27 | 3 | 11 |
| 12 | What are the best ways to provide timely and appropriate information about palliative care to children and young people with cancer, and their families, and to support their understanding of care options? | 32 | 36 | 12 |
| 13 | How can children and young people with cancer from regional, rural and remote locations of Australia, and their families, access better support services? (e.g. when travelling for treatment, living away from home or seeking services) | 8 | 13 | 13 |
| 14 | How can problems with memory, learning and literacy be better identified, prevented and managed in children and young people with cancer? ​ | 32 | 19 | 14 |
| 15 | How can hospital environments and experiences be improved for children and young people with cancer to better meet their unique physical, developmental and emotional needs? | 29 | 7 | 15 |
| 16 | How can complementary and natural therapies be integrated in contemporary cancer care for children and young people?​ | 36 | 33 | 16 |
| 17 | What socio-economic, geographic, and logistical barriers and enablers in Australia affect access to novel cancer treatments and clinical trials for children and young people with cancer? | 48 | 31 | 17 |
| 18 | What are the best ways to address the social and emotional health needs of children and young people during and after cancer treatment? | 27 | 8 | 18 |
| 19 | How can a more personalised and tailored approach to childhood cancer treatment be developed and delivered to meet the unique needs of each person? | 6 | 19 | 18 |
| 20 | How can access to holistic supportive care services be improved for children and young people with cancer, both during and after treatment? | 25 | 16 | 20 |
| 21 | How can screening tests, germline genetic sequencing, and personalised medicine be used more widely to predict the risk of cancer in children and young people, find it early and guide treatment choices? | 5 | 24 | 21 |
| 22 | What are the best ways to ensure equitable and timely access to information about fertility preservation for children and young people diagnosed with cancer | 36 | 24 | 21 |
| 23 | What are the barriers to prompt detection and accurate diagnosis of cancer in children and young people?​ | 41 | 19 | 23 |
| 24 | How can quality of life and symptom management be improved for children and young people with cancer who experience severe, persistent pain? | 44 | 29 | 23 |
| 25 | What are the best ways and when is the best time to give information about survivorship and the long-term effects of treatment to people affected by childhood cancer? | 19 | 4 | 25 |
| 26 | How does continuity of care during and after cancer treatment for children and young people affect their long-term psychosocial well-being and healthcare utilisation? What models of care can support this? | 21 | 18 | 26 |
| 27 | What are the best ways to give families information about childhood cancer support services and what processes support and empower families to seek help? | 31 | 24 | 26 |
| 28 | How can shared and informed decision making about childhood cancer treatment and follow-up care be supported and improved? | 25 | 24 | 28 |
| 29 | What are the best ways to coordinate multidisciplinary cancer care for children and young people to ensure families have clear, consistent support within hospitals and in the community?​ | 21 | 32 | 29 |
| 30 | What are the best ways to foster peer support between children or young people with cancer and their carers? ​ | 44 | 19 | 30 |
| 31 | How can support services be tailored to address the unique needs of different family members, particularly siblings, during and after a child’s cancer treatment? | 23 | 29 | 30 |
| 32 | What effect do educational interventions have on the education outcomes of children and young people treated for cancer? | 36 | 10 | 32 |
| 33 | What causes cancer in children and young people? (e.g. genetic variations, parental exposure to toxins in pregnancy or the environment) | 47 | 13 | 33 |
| 34 | What are the effects of physical activity, nutrition and sleep on health outcomes during and after treatment for cancer in children and young people? | 4 | 36 | 33 |
| 35 | What factors contribute to the risk of cancer relapse or treatment resistance in children and young people? Can identifying these factors early help personalise treatment to improve outcomes? ​ | 8 | 19 | 35 |
| 36 | What resources are needed to make complex information about the role of genetics and gene mutations accessible to children and young people with cancer and their families? | 11 | 35 | 36 |
| 37 | What strategies would improve medical, nursing and allied health professionals' training to ensure they are equipped to provide comprehensive cancer care to children and young people with cancer in hospitals and community settings? | 17 | 45 | 36 |
| 38 | How can equitable and effective long-term follow-up after treatment for childhood cancer be implemented in Australia? | 15 | 12 | 38 |
| 39 | How can cancer care be improved for children and young people who have co-occurring psychosocial or developmental conditions (such as ASD, ADHD, and learning disorders)? | 14 | 48 | 39 |
| 40 | Can cancer in children and young people be prevented? | 49 | 16 | 40 |
| 41 | What are the best ways to provide palliative care for children and young people with cancer in regional, rural and remote locations in Australia? | 35 | 45 | 40 |
| 42 | What are the barriers and enablers in Australia to delivering cancer treatment to children and young people as close to home as possible? | 44 | 36 | 42 |
| 43 | What are the best ways to provide tailored and accessible information about cancer in children and young people, its treatment and side effects? | 36 | 6 | 43 |
| 44 | How can the needs of children and young people with cancer in vulnerable social circumstances (e.g. limited family support) be better identified and managed?​ | 32 | 39 | 43 |
| 45 | What is the effect of cancer survivorship programs for children and young people on health outcomes and healthcare costs? | 2 | 42 | 45 |
| 46 | How can health and community services better engage with First Nations children and young people with cancer, their kin and families to provide culturally safe care and support services? | 10 | 48 | 46 |
| 47 | What are the best ways to support and retain health and community care professionals who provide cancer care to children and young people, across all areas of Australia? | 43 | 39 | 47 |
| 48 | What strategies would support early integration of palliative care alongside active treatment for children and young people with difficult-to-treat cancer? | 13 | 42 | 47 |
| 49 | What are the barriers and enablers for families in accessing grief support services after the loss of a child from cancer? | 42 | 45 | 49 |

*H; Health/social professionals P; Patients/survivors C; Caregivers*

## **Appendix C: Workshop final small group rankings and collective overall rankings**

| **Rank** | **Question** | **G1** | **G2** | **G3** |
| --- | --- | --- | --- | --- |
| **1** | How can the development of cancer treatments and equitable access to treatment be optimised for children and young people so that treatments are safer, less toxic, less traumatic, and more effective? | 1 | 1 | 1 |
| **2** | What are the best ways to prevent, identify and manage the long-term effects of cancer and its treatment in children and young people on physical and psychological health? | 2 | 2 | 2 |
| **3** | What are the best ways to support children and young people through transitions from active cancer treatment to follow-up and everyday life? (e.g., re-engagement with education and communities and transition to adult healthcare)? | 4 | 4 | 4 |
| **4** | What is the effect of cancer survivorship programs for children and young people on health outcomes and healthcare costs? | 5 | 5 | 6 |
| **5** | How can psychosocial interventions that prevent or minimise the trauma of cancer treatment in children and young people be better integrated to care? | 3 | 3 | 14 |
| **6** | How can health and community services better engage with First Nations children and young people with cancer, their kin and families to provide culturally safe care and support services? | 7 | 9 | 9 |
| **7** | How can a more personalised and tailored approach to childhood cancer treatment be developed and delivered to meet the unique needs of each person? | 6 | 12 | 8 |
| **8** | How can children and young people with cancer from regional, rural and remote locations of Australia, and their families, access better support services? (e.g. when travelling for treatment, living away from home or seeking services) | 15 | 6 | 7 |
| **9** | What are the psychosocial effects of cancer in children and young people, and their families, during and after treatment, and how can these effects be addressed? | 8 | 17 | 3 |
| **10** | What factors contribute to the risk of cancer relapse or treatment resistance in children and young people? Can identifying these factors early help personalise treatment to improve outcomes? | 13 | 7 | 10 |
| **11** | How can hospital environments and experiences be improved for children and young people with cancer to better meet their unique physical, developmental and emotional needs? | 10 | 16 | 5 |
| **12** | What are the best ways to predict, reduce and manage the short and long-term side effects of cancer treatment in children and young people? | 9 | 11 | 15 |
| **13** | How can the needs of families be best identified and addressed when a child's cancer is at risk of or does relapse? | 12 | 13 | 12 |
| **14** | What are the best ways to provide tailored and accessible information about cancer in children and young people, its treatment and side effects? | 11 | 10 | 17 |
| **15** | How can screening tests, germline genetic sequencing, and personalised medicine be used more widely to predict the risk of cancer in children and young people, find it early and guide treatment choices? | 17 | 8 | 16 |
| **16** | What are the long-term effects of different cancer diagnoses and different treatments on a child’s cognition, education and social health? | 16 | 15 | 13 |
| **17** | What are the best ways to provide social and emotional support to family caregivers of children and young people diagnosed with cancer, during and after treatment and into survivorship? | 14 | 14 | 18 |
| **18** | What are the effects of physical activity, nutrition and sleep on health outcomes during and after treatment for cancer in children and young people? | 18 | 18 | 11 |
| **19** | What are the best ways and when is the best time to give information about survivorship and the long-term effects of treatment to people affected by childhood cancer? | 19 | 19 | 19 |

*Note. Each small group (G) consisted of 9 participants with representation from childhood cancer survivors, caregivers and health professionals. The overall ranking was obtained by averaging the small group ranking, with a lower number rank indicating a higher priority. In cases where two uncertainties received the same average ranking, the final order was determined using the geometric mean*
